# Transition from sub-Rayleigh anticrack to supershear crack propagation in snow avalanches

**DOI:** 10.1038/s41567-022-01662-4

**Published:** 2022-07-25

**Authors:** Bertil Trottet, Ron Simenhois, Gregoire Bobillier, Bastian Bergfeld, Alec van Herwijnen, Chenfanfu Jiang, Johan Gaume

**Affiliations:** 1 École Polytechnique Fédérale de Lausanne, Lausanne, Switzerland; 2 Colorado Avalanche Information Center, Boulder, CO USA; 3 WSL Institute for Snow and Avalanche Research SLF, Davos, Switzerland; 4 Department of Mathematics, University of California, Los Angeles, CA USA

**Keywords:** Applied physics, Computational science, Soft materials

## Abstract

Snow slab avalanches, characterized by a distinct, broad fracture line, are released following anticrack propagation in highly porous weak snow layers buried below cohesive slabs. The anticrack mechanism is driven by the volumetric collapse of the weak layer, which leads to the closure of crack faces and to the onset of frictional contact. Here, on the basis of snow fracture experiments, full-scale avalanche measurements and numerical simulations, we report the existence of a transition from sub-Rayleigh anticrack to supershear crack propagation. This transition follows the Burridge–Andrews mechanism, in which a supershear daughter crack nucleates ahead of the main fracture front and eventually propagates faster than the shear wave speed. Furthermore, we show that the supershear propagation regime can exist even if the shear-to-normal stress ratio is lower than the static friction coefficient as a result of the loss of frictional resistance during collapse. This finding shows that snow slab avalanches have fundamental similarities with strike-slip earthquakes.

## Abstract (web summary)

Avalanches can occur when a porous snow layer lies beneath a dense cohesive snow slab. Field experiments and simulations now reveal different crack-propagation regimes in slab avalanches, similar to rupture propagation following an earthquake.

## Main

The release of snow slab avalanches results from a succession of mechanical processes^1,2^. A failure is initiated in a highly porous weak snow layer buried beneath a cohesive snow slab, leading to mixed-mode and quasi-brittle crack propagation along the slope^3^. If the slope angle is larger than the weak layer friction angle, the slab eventually slides and releases^4^.

Two seemingly conflicting theories have been developed to conceptualize avalanche release mechanisms. The first was introduced by McClung^1^ and assumes that the weak layer fails under shear only (mode II). By construction, this approach relies on the slope-parallel component of the load and thus fails to explain field observations of crack propagation on flat terrain and remote avalanche triggering^5–7^. A second theory was thus proposed by Heierli et al.^8^, who extended the concept of anticracks originally introduced to explain deep earthquakes^9^ to slab avalanches. In contrast to classical cracks opening under tension (mode I), anticracks refer to a mode of compressive fracture with the closure of crack faces (mode −I). This process thus requires a volume reduction (collapse), which is only possible in porous materials under pressure. In this theory^8^, the volumetric collapse of the weak layer allows the overlaying slab to bend, which induces stress concentrations in the weak layer and drives anticrack propagation, even on low-angle slopes. Despite more recent advances reconciling these two approaches^10^, the dynamic phase of crack propagation prior to frictional sliding remains largely unknown, especially at the slope scale. Very large crack speeds (>150 m s^−1^) were evaluated based on real-scale avalanche observations by Hamre and others^11^. Although these findings only represent indirect measurements and lack validation, the reported values appear larger than the shear wave speed in snow, which contrasts with the substantially lower sub-Rayleigh values measured in small-scale snow fracture experiments^12^. This discrepancy between small-scale experiments and large-scale observations suggests that there could be a transition during fracture propagation.

Crack speeds greater than the shear wave speed *c*_s_, but less than the longitudinal wave speed *c*_p_, have been reported in earthquake dynamics based on seismological observations^13–15^, stick-slip^16–18^ and fracture experiments^19,20^, and numerical simulations^15,21,22^. This complex process, at the frontier between fracture and friction, is often referred to as intersonic or supershear crack propagation. It has been conceptualized by Burridge–Andrews^23,24^, who showed that a daughter supershear crack can nucleate ahead of the main fracture if the local shear stress exceeds a critical value. Although snow slab avalanches have numerous mechanical features in common with earthquakes, the snow anticrack mechanism, which allows for crack propagation without an external driving shear force, further complicates analyses and prevents direct analogies.

In this Article, based on numerical simulations, snow fracture experiments and full-scale avalanche measurements, we show that, under specific conditions, the onset of frictional sliding during slab avalanche release may involve a transition from sub-Rayleigh anticrack to supershear crack propagation. This transition implies the Burridge–Andrews mechanism and can occur even for slope angles smaller than the weak layer friction angle; we attribute this to the loss of frictional resistance during weak layer collapse related to the anticrack. We quantify the so-called super-critical crack length associated with this supershear transition and propose a theoretical shear band model in which the collapse amplitude is accounted for by a reduced effective friction.

### Experimental mismatch

**Extended Data Fig. 1:**
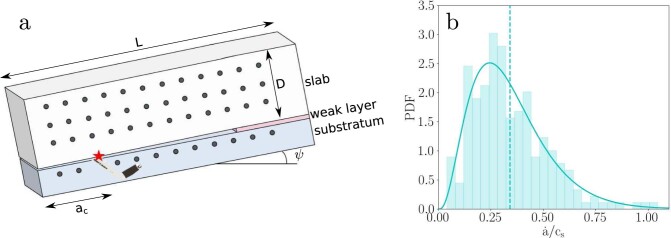
Propagation Saw Test setup and measured crack propagation speeds. **a**, Schematic of the Propagation Saw Test. **b**, Histogram of the normalized crack propagation speeds obtained from 222 PSTs following a beta law probability density distribution (solid line). The dashed line corresponds to the median of the distribution.

Based on elastic modulus values measured using snow fracture tests^25^, the effective shear wave speed *c*_s_ in snow is less than 120 m s^−1^. Crack propagation speeds obtained based on numerous small-scale (<2 m) snow fracture experiments typically vary between 10 m s^−1^ and 80 m s^−1^ (Extended Data Fig. 1). However, Hamre et al.^11^ reported crack propagation speeds larger than 200 m s^−1^, based on slope-scale avalanche observations, suggesting supershear fracture. To explain these contrasting observations, the dynamics of crack propagation in weak snow layers is analysed via numerical simulations, snow fracture experiments and full-scale avalanche measurements.

### Numerical simulations

**Fig. 1:**
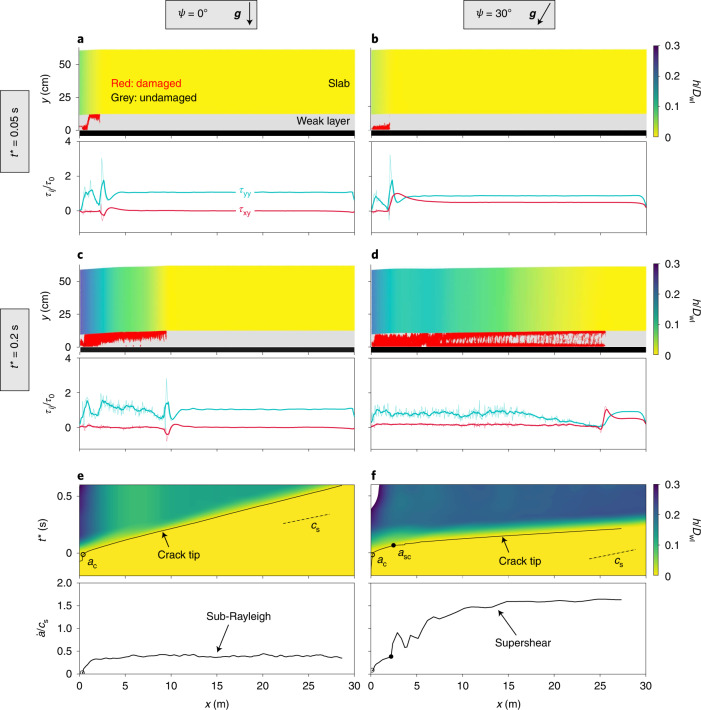
Crack tip morphology, dynamics and stress state for slopes of 0° and 30°. **a**,**b**, Top: weak layer fracture (in red) and slab vertical displacement *h* scaled by weak-layer thickness *D*_wl_ for *ψ* = 0° (**a**) and *ψ* = 30° (**b**) Bottom: shear (red) and normal (green) stress profiles in the weak layer. t* = *t* − *t*_c_ = 0.05 s (*t*_c_ is the time corresponding to the onset of crack propagation). **c**,**d**, As in **a**,**b**, for t* = 0.2 s. **e**,**f**, Top: spatio-temporal evolution of *h*/*D*_wl_ and crack tip location for *ψ* = 0° (**e**) and *ψ* = 30° (**f**). Black solid lines, crack tip position; open circles, critical crack length *a*_c_; filled circles, super-critical crack length *a*_sc_. Bottom: crack propagation speed.

**Fig. 2:**
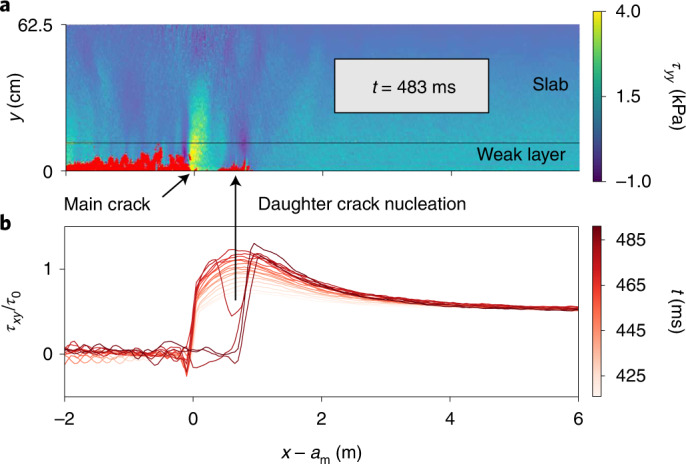
Illustration of the Burridge–Andrews mechanism leading to onset of the supershear crack propagation regime in the simulation shown in Fig. 1 for a slope of 30°. **a**, Visualization of the instant of the supershear transition showing the birth of a daughter crack in front of the main crack. Cracks are shown in red and the system is additionally coloured by the normal stress *τ*_*yy*_. **b**, Evolution of the shear stress *τ*_*xy*_ normalized by the nominal stress *τ*_0_ as a function of *x* − *a*_m_ (*x*, longitudinal position; *t*, time; *a*_m_, position of the main crack).

Numerical simulations of the so-called propagation saw test (PST)^6^ were performed based on the material point method (MPM), finite strain elastoplasticity and a constitutive snow model accounting for weak-layer volumetric collapse^3^ (Methods). The PST consists of creating a crack in a predefined weak layer using a saw, until it reaches a critical crack length *a*_c_ (corresponding to time *t*_c_), after which the crack propagates in a self-sustained manner. The main differences between snow fracture experiments and real avalanches are the length scale and slope angle (*ψ*), which are generally low in experiments for practical and safety reasons. Accordingly, simulations were performed for very long PSTs, up to 140 m, and for representative slope angles between 0° and 50°. Figure 1a–d shows weak-layer fracture and the vertical displacement of the slab, *h* (scaled by weak-layer thickness *D*_wl_), for flat and 30° slopes, respectively (Supplementary Video 1) at two different times t* = *t* − *t*_c_. Based on the spatio-temporal evolution of the crack tip, the crack propagation speed can be evaluated (Fig. 1e,f). On a flat surface, slab bending induced by weak-layer collapse drives anticrack propagation. After reaching the critical crack length, the crack jumps at the slab–weak layer interface, and the propagation speed increases until it reaches a sub-Rayleigh plateau value. Conversely, on an inclined slope, the slow increase in the crack speed is followed by a sharp transition, leading to different crack propagation dynamics than observed on flat terrain. This transition occurs for a ‘super-critical crack length’ *a*_sc_, which is significantly larger than *a*_c_, and starts with nucleation of a daughter crack in front of the main fracture (Fig. 2). This topological change results in an apparent jerk in the crack length followed by a sharp increase in the crack speed to a supershear regime that then converges to a speed of ${1.6}{{c}_{\rm{s}}}\approx {\sqrt{2({1}+{\nu })}}{{c}_{\rm{s}}}={\sqrt{{E}/{\rho }}}$, where *E*, *ν* and *ρ* are the slab elastic modulus, Poisson’s ratio and density, respectively. Note that, in the analysis of our simulations and the evaluation of the crack speed, the hypothesis of crack uniqueness is made. Accordingly, the crack tip is defined as the location of the furthest plasticized particle. Based on our single crack tip definition, the Burridge–Andrews mechanism necessarily implies an infinite propagation speed after reaching *a*_sc_, which explains the reported sudden jerk. In principle, and as described by Liu and Lapusta^26^, the daughter crack (Fig. 2) spontaneously propagates as a supershear crack in both directions and then merges with the main crack. Once supershear propagation occurs, the crack spontaneously branches, leading to complex micro-crack patterns, as reported by Sharon and others^27^. We also verified that the secondary parallel fracture at the interface with the bed surface appears in all supershear simulations. This is directly related to the rigid substratum and disappears when the substratum is soft (deformable). Note that we performed simulations to confirm that the stiffness of the substratum does not affect the existence and speed of the supershear crack propagation regime.

### Onset of supershear fracture

**Fig. 3:**
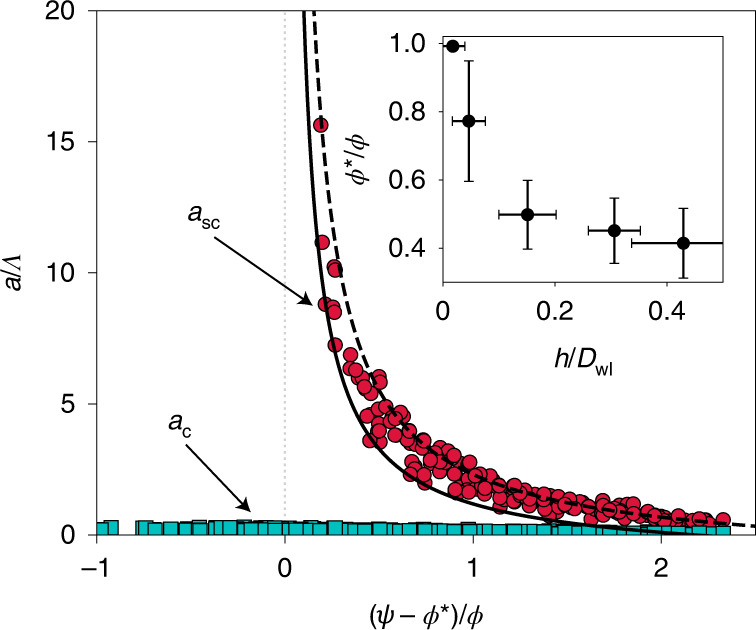
Simulation of critical and super-critical crack lengths normalized by the elastic length *Λ* as a function of the normalized slope angle. A simulation is presented of critical (green squares) and super-critical (red circles) crack lengths normalized by the elastic length *Λ* as a function of the normalized slope angle $(\psi-\phi^{*})/\phi$ (*ϕ*, friction angle; $\phi^{*}$, effective friction angle). *S*olid and dashed lines correspond to theoretical curves for effective friction angles of $\phi^{*}=\phi$ and $\phi^{*}=0.4$, respectively. The inset shows $\phi^{*}/\phi$ as a function of the collapse amplitude *h* normalized by weak-layer thickness *D*_wl_ (data points correspond to an average of several simulations and error bars represent the s.d.).

**Extended Data Fig. 2:**
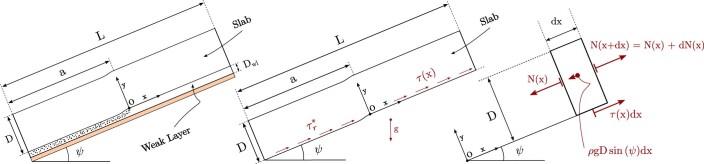
Illustration of the system and force balance related to the analytic model for the onset of supershear propagation. Notations are described in the Methods section.

Crucial insights with respect to the mechanical origin of this transition are found by examining the stress state in the weak layer (Fig. 1a–d). On flat terrain, bending of the slab induces stress concentrations at the crack tip and a negative shear stress (directed up-slope). At the crack tip, the normal stress, higher than the shear stress, drives anticrack propagation. The stress profile in the vicinity of the crack tip remains approximately self-similar throughout the entire simulation (Fig. 1a,c). Behind the crack, the normal stress fluctuates around the far-field value, corresponding to the new contacts between the crack faces. On the slope, slab tension induced by the slope-parallel component of the gravitational force overcomes the stresses induced by slab bending, leading to a positive shear stress peak at the crack tip (directed down-slope). Shear and normal stress are initially of the same order of magnitude, and both drive mixed-mode anticrack propagation. After reaching the super-critical crack length, a clear and sharp transition of the stress profile in the vicinity of the crack tip is observed (Figs. 1b,d and 2b). The normal stress becomes very low, and the crack is driven by shear. Therefore, the transition from sub-Rayleigh to supershear crack speeds is always accompanied by a transition from mixed-mode anticrack to pure mode-II crack propagation. Under these conditions, the super-critical crack length can be described by a modified shear band propagation model under the effect of strain softening^28,29^ and weak-layer collapse (Methods and Extended Data Fig. 2):

1 $${a}_{\rm{sc}}={{\varLambda }{}\frac{{{\tau }_{\rm{p}}}-{{\tau }_{\rm{g}}}}{{{\tau }_{\rm{g}}}-{{\tau }_{\rm{r}}^{* }}}},$$

where *Λ* is a characteristic elastic length related to the elasticity and height of the two layers, *τ*_p_ is the weak-layer shear strength, *τ*_g_ *=* *τ*_0_sin*ψ* is the shear stress induced by the slab nominal load *τ*_0_, and ${{\tau }_{\rm{r}}^{* }}={{{\tau }_{0}}\cos \psi \tan {\phi }^{* }}$ is the shear band effective residual stress and is a function of both the slab load and the effective friction angle ${\phi }^{* }$, which depends on the weak-layer friction angle and the collapse height (Fig. 3). It should be noted that our super-critical crack length *a*_sc_ shows similarities with the length proposed in previous studies on slip instabilities (same trend with slope angle and divergence close to the friction angle, as in refs. ^24^ and ^21^). Yet, the bilayer configuration studied here prevents a direct analogy between these different formulations.

### Collapse-dependent friction weakening

Numerous numerical simulations were performed for different slope angles and mechanical properties of both the slab and weak layer (Methods) to evaluate the condition for the onset of anticrack (*a*_c_) and supershear (*a*_sc_) propagation. The critical crack length decreases with increasing slope angle *ψ* and is on the order of 0.1*Λ* (Fig. 3). The super-critical crack length only exists if the slope angle *ψ* is larger than the effective friction angle ${\phi }^{* }$. It also decreases with increasing slope angle and varies between 0.5*Λ* and 15*Λ* in the simulations. The effective friction coefficient controls the onset of the supershear transition and significantly depends on the collapse amplitude *h* of the weak layer (Fig. 3, inset). Without volumetric collapse, the effective friction angle is exactly equal to the friction angle. However, increasing collapse heights reduce the effective frictional resistance of the shear band, as reported in ref. ^4^. This local friction reduction enables a supershear transition for slope angles lower than the weak-layer friction angle. In effect, once the crack reaches its super-critical crack length, its sharp acceleration is associated with a significant increase of the slab section that is not supported by the weak layer, leading to unstable propagation even below the friction angle. The simulation data are well reproduced by equation (1) for an effective friction angle between 0.4*ϕ* and *ϕ*.

### Propagation speed regimes and validation

**Fig. 4:**
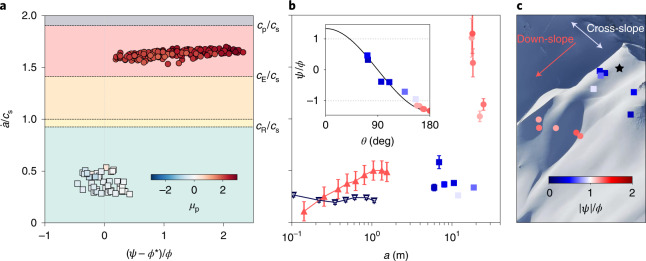
Crack propagation speed in numerical simulations, field experiments and full-scale measurements. **a**, Asymptotic crack speeds obtained in numerical simulations. Points are coloured according to the peak shear-to-normal stress ratio *μ*_p_. The background colour represents different propagation regimes limited by the Rayleigh wave speed *c*_R_, the shear wave speed *c*_s_, the Eshelby wave speed ${{c}_{\rm{E}}}={\sqrt{2}}{{c}_{\rm{s}}}$ and the p-wave speed *c*_p_. **b**, Crack speeds obtained in field experiments (flat PST, dark blue triangles; 37° PST, red triangles) and full-scale avalanche measurements (cross-slope speed, blue squares; down-slope speed, red circles). The determination of error bars is described in the Methods. The inset represents the normalized slope angle versus the azimuthal angle *θ*, characterizing crack direction (down-slope or cross-slope). **c**, Location of the measurement points on the slope prior to the avalanche.

The asymptotic crack propagation speeds obtained in all simulations are shown in Fig. 4a. For slope angles lower than the effective friction coefficient, the asymptotic propagation speed is sub-Rayleigh and varies between 0.25*c*_s_ and 0.6*c*_s_. In this regime, the peak shear-to-normal stress ratio, *μ*_p_, is low due to large normal stresses at the peak (Fig. 1c) and changes in sign according to the shear stress nature (that is, negative when slab bending is dominant and positive when slab tension is dominant). For larger slope angles, the propagation becomes supershear with a speed approaching a value of $1.6{{c}_{\rm{s}}} \sim {\sqrt{{E}/{\rho }}}$, similar to the longitudinal elastic wave speed in a beam. In this case, the large values of *μ*_p_ indicate that this regime is driven by large shear stress and low normal stress values (Figs. 1d and 2) resulting from slab tension.

**Extended Data Fig. 3:**
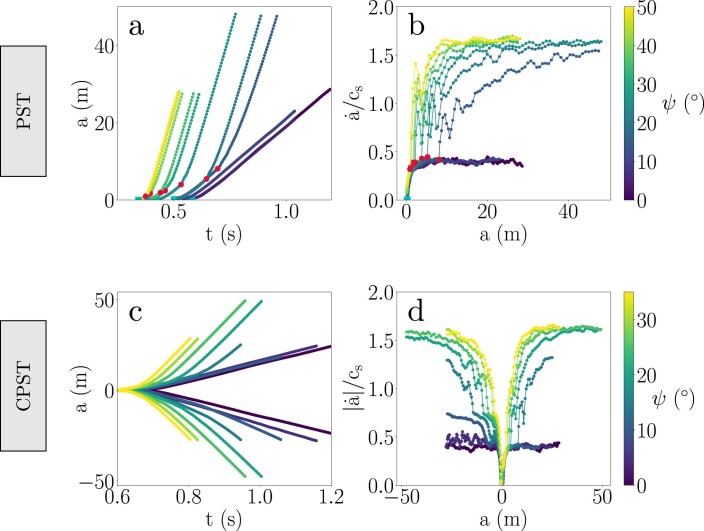
Effect of slope angle on the propagation dynamics in standard Propagation Saw Tests (PSTs, top) and Centered Propagation Saw Tests (CPSTs, bottom). **a**, Temporal evolution of the crack tip location. Blue squares and red circles correspond respectively to critical and super critical crack lengths. **b**, Normalized crack propagation speed as a function of slope angle. The transition to the supershear regime requires a smaller super critical crack length for larger slope angles. A steeper slope induces a shorter transient regime. **c**, Temporal evolution of the crack tip location. **d**, Normalized crack propagation speed as a function of the crack length for different values of slope angle. This CPST setup induces propagation in both upslope and downslope directions. While the complex interplay between slab bending induced by weak layer collapse and slab tension (upslope) or slab compression (downslope) breaks the propagation symmetry, the asymptotic speeds are essentially the same in both directions.

**Extended Data Fig. 4:**
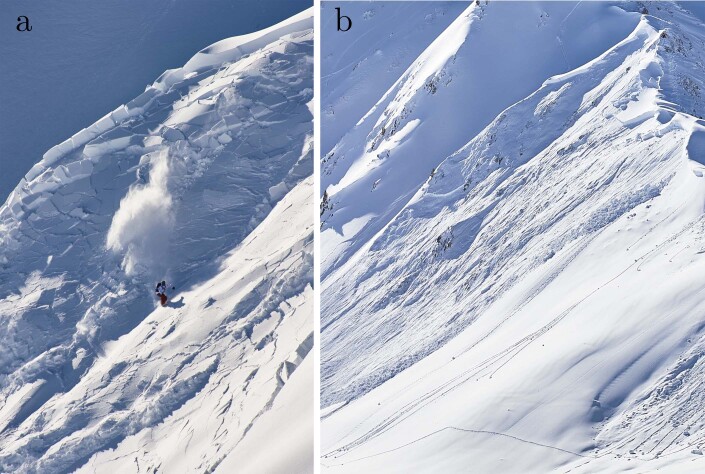
Supershear snow slab avalanche after the release. **a**, Snowboarder successfully escaping the release zone after triggering the avalanche. **b**, Situation of the slope after the avalanche. Photos: Ruedi Flück.

**Extended Data Fig. 5:**
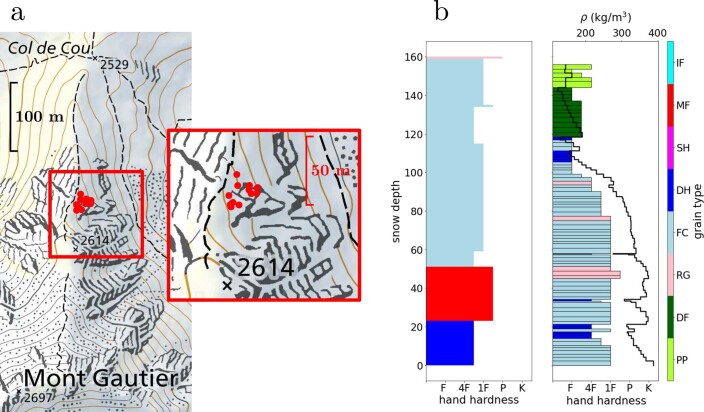
Slope and snowpack characteristics. **a**, Location of the avalanche site on the geo.admin.ch portal and position of key-points determined based on the video analysis method. **b**, Manual snow profile (left) and SNOWPACK simulation (right) showing density and grain size of the different layers.

**Extended Data Fig. 6:**
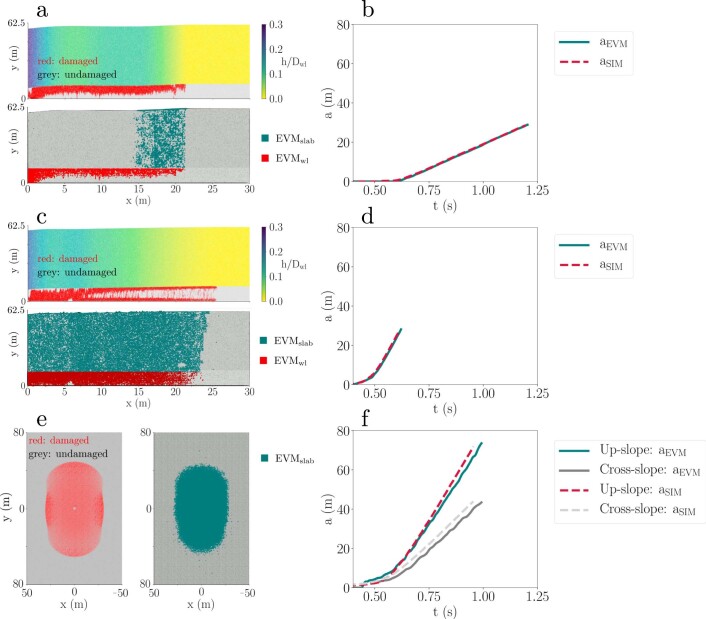
Validation of the Eulerian Video Magnification (EVM) method based on MPM simulations. **a**, Application of the EVM method on a 2D PST simulation on flat terrain. The top left panel shows the simulation result and the bottom left panel shows the EVM result for one selected frame. The plot on the right shows the crack tip position as a function of time based on the simulation analysis and the EVM method, showing a good agreement. **b**, same as **a** for a 30 degrees slope. **c**, Application of the EVM method on a 3D MPM simulation of a slab - weak layer system on a 30 degrees slope. In this simulation the radius of a circular crack in the weak layer is increased until the crack propagates. The left panel shows the weak layer failure state (top view) and the middle panel shows the EVM analysis based on slab deformation (top view). The EVM method gives a good estimate of the crack propagation speed both in the up-slope and cross slope directions.

**Extended Data Fig. 7:**
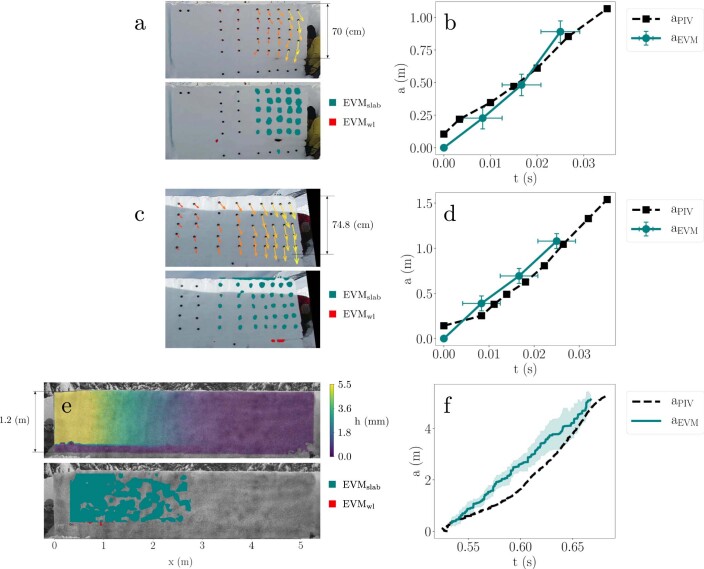
Validation of the Eulerian Video Magnification (EVM) method based on PST experiments. **a**, Application of the EVM method on the PST experiment on flat terrain presented in the paper. The top left panel shows the result of the Particle Image Velocimetry (PIV) analysis. The arrows length and color represent the slab displacement direction and magnitude, respectively. The bottom left panel shows the EVM result for one selected frame. The plot on the right shows the crack tip position as a function of time based on the PIV analysis and the EVM method, showing a good agreement. **b**, same as **a** for a the 37 degrees PST presented in the paper. **c**, same as **a** for a 5 m long PST experiment on flat terrain. On the top left panel, the slab is colored by the slab vertical displacement *h*.

Crack propagation speeds obtained in two representative snow fracture experiments (Methods and Extended Data Table 1) performed on flat terrain and on a 30° slope indicate sub-Rayleigh propagation with speeds (Fig. 4b) in line with those obtained in the numerical simulations in this regime. For the experiments on flat terrain, the bending of the slab is limited by the collapse amplitude, which leads to a constant crack propagation speed. However, for the inclined experiment, slab tension increases during crack propagation, leading to an increase in the crack speed. This is corroborated by our numerical simulations (Extended Data Fig. 3). The sub-Rayleigh speed obtained in the high-angle experiment highlights that this transition requires both a steep slope and a crack length that reaches *a*_sc_. In this tilted experiment, based on equation (1) and the reported relative collapse amplitude of 6%, *a*_sc_ is larger than the beam length typically used in this test (~1.5 m). As a consequence, classical snow fracture experiments (that is, PSTs^25,30^) are not well suited to characterize this transition, which requires slope-scale dimensions. An analysis of a full-scale deep slab avalanche (Extended Data Figs. 4 and 5) triggered in 2019 in Wallis, Switzerland, on a 42° slope allows us to experimentally confirm the existence of a supershear propagation regime and to further characterize crucial spatial characteristics. Based on an analysis of the change in frame pixel intensity (Methods, Supplementary Videos 2 and 3 and Extended Data Figs. 6 and 7), the crack propagation speeds can be measured at different key points (Fig. 4b,c) in directions varying between down-slope and cross-slope (characterized by the azimuthal angle *θ*). Crack speeds in the down-slope direction (mode II) are supershear, with values in good agreement with the asymptotic values obtained in steep and long numerical PSTs (Extended Data Fig. 3). Conversely, cross-slope (mode III) and sub-Rayleigh speeds are in good agreement with the asymptotic values obtained in low-angle numerical simulations, as well as experimental PSTs.

### Discussion

**Extended Data Fig. 8:**
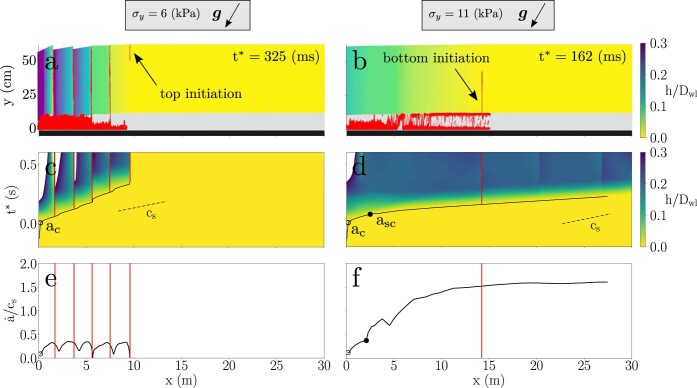
Effect of slab tensile failure on supershear crack propagation. Outcome of the simulation shown in Fig. 1b, (supershear transition for a 30^∘^ slope) in which we account for slab tensile failure for a relatively soft (*σ*_t_ = 6 kPa, left) and hard (*σ*_t_ = 11 kPa, right) slab. **a, b**: PST colored by normalized vertical displacement (slab) and weak layer fracture (in red) at two different times. **c, d**: spatio-temporal evolution of *h*/*D*_*wl*_ and crack tip location. Black solid lines: crack tip position; empty circles: critical crack length *a*_*c*_; filled circles: super critical crack length *a*_*sc*_. Red lines: slab fracture. **e, f**: normalized propagation speed. This figure shows that the tensile failure of soft slab can prevent the supershear transition (panels **a** and **c**). In this case, the volumetric collapse of the weak layer drives anticrack propagation because the slab cannot accumulate the necessary amount of strain energy required for supershear fracture. This leads to the so-called ‘en-echelon’ type of fracture^6,16^. The crack propagation speed in this case is varying within a sub-Rayleigh range (panel **e**). The propagation speed decreases locally after the onset of slab tensile failure. Eventually, crack propagation is stopped after an ultimate slab fracture. In contrast, for harder slabs, slab tensile failure may occur but does not perturb crack propagation dynamics and supershear fracture (panels **b** and **d**). In that case, the crack propagation speed is almost identical to that obtain for an elastic slab (Fig. 1b).

In our simulations, the slab was considered purely elastic. By accounting for slab tensile failure, we show (Extended Data Fig. 8) that soft slabs with low tensile strengths prevent the transition from occurring. This leads to slab fracture that starts from the snowpack surface, where the tensile stress is the highest, and occasionally to the so-called ‘en-echelon’ characteristic. In this case, the propagation is substantially affected by slab fracture, leading to crack arrest and therefore to small release areas. For denser slabs with higher tensile strength, slab fractures do not significantly affect crack propagation, and the occurrence of the supershear transition. Release areas can therefore be much larger, and probably mostly constrained by topography (for example, changes in slope angle). In this situation, slab fracture starts from the bottom at the slab–weak layer interface where tension is largest in the absence of slab bending (Fig. 1d). These implications of our results are consistent with observations of (1) slab fractures initiated at the surface in small-scale fracture experiments^31^ and indirect observations of slab fracture initiated at the bottom of snowpacks in real avalanches^32^ and (2) soft slab avalanches typically being smaller than hard ones^7,33^.

As a consequence, it is expected that large slab avalanches, usually involving hard slabs^33^, would be supershear. This finding has important consequences for hazard management in mountainous regions, which still relies on empirical evaluations of the avalanche release size. The new paradigm reported here implies that weak-layer collapse and slab bending do not influence the dynamics of supershear cracks. Hence, a simple shear model can be sufficient to predict the sizes of large slab avalanches typically larger than hundreds of metres^34^ by neglecting the anticrack propagation regime, which occurs over distances shorter than ~3–5 m. Therefore, considering a depth-averaged model with a shear interface for the weak layer would allow the present work to be brought to an operational level, improving avalanche risk assessment and forecasting.

Furthermore, our findings indicate that the crack propagation speed measured in small-scale experiments is not necessarily representative of crack speeds on real avalanche terrain in the down/up-slope direction (mode II). In fact, despite the different propagation mechanisms, the experimentally measured values are in good agreement with the avalanche cross-slope propagation speeds (Fig. 4b, mode III), which are theoretically limited by the Rayleigh wave speed^23^. Here we provide a two-dimensional (2D) theoretical framework that allows the conditions for the onset of this transition to be evaluated, as well as the crack propagation speed, which depends on slab elastic waves. Future work should include slope-scale experiments and simulations to study 3D propagation patterns^35,36^, as well as the complex interplay between the weak layer and slab fracture during the release process.

Our findings shed light on a previously unreported stage of the avalanche release process, with key implications for predictions of avalanche danger. More generally, our results reinforce the analogy between snow slab avalanches and earthquakes. Although the mechanism of supershear propagation has rarely been reported in large strike-slip earthquakes^15^, it requires a very common combination of topographical and mechanical ingredients in slab avalanche release.

## Methods

### Snow fracture experiments

A total of 222 snow fracture experiments (so-called PSTs) were conducted in Davos, Switzerland during winter of 2015–2016. These experiments followed the standards defined in ref. ^30^. Each experiment was recorded with a high-speed camera, allowing us to compute the displacement of black markers inserted in the pit wall using particle tracking velocimetry^12^ with an accuracy of ~0.1 mm. Crack propagation velocity was measured based on the temporal delay between the onset of vertical motion of the markers based on the average speed computed from six vertical displacement thresholds (0.07, 0.08, 0.09, 1.0, 1.1 and 1.2 mm). Two PSTs from this large dataset were selected for in-depth analysis. Both experiments have similar mechanical and geometrical properties, as presented in Extended Data Table 1. The standard deviations of PSTs presented in Fig. 4 were computed from the mean speed deviation based on all thresholds. The main difference between the experiments was the slope angle, as one experiment was performed on flat terrain and the other on a 37° slope. Crack propagation speeds obtained in these two experiments (${\dot{a}}/{{c}_{\rm{s}}} \approx {0.2}$ in PST 1 and ${\dot{a}}/{{c}_{\rm{s}}} \approx {0.4}$ in PST 2, where ${\dot{a}}$ is the crack propagation speed) are representative of those obtained in the 222 PSTs, as shown in Extended Data Fig. 1b.

### Full-scale avalanche measurements

On 31 January 2019, a professional snowboarder triggered a dry-snow slab avalanche (Extended Data Fig. 4a) in a location near Col du Cou in Wallis, Switzerland (Extended Data Fig. 5a). A few minutes previously, the group had checked the snowpack stability on a slope immediately behind and did not trigger an avalanche (see ski tracks in Extended Data Fig. 4b). The snowboarder triggered this slab avalanche because of the large impact force induced by a jump from the ridge (Supplementary Video 3), which led to failure initiation and crack propagation in the buried weak snow layer close to the ground.

The avalanche was recorded using a high-quality video camera (with a frame rate of 50 frames per second), allowing analysis of slab motion induced by crack propagation within the buried weak snow layer. Our crack propagation speed measurements rely on four stages: (1) video stabilization using optical flow, (2) Eulerian video magnification (EVM) to enhance small changes in snow reflection due to slab deformation, and detection of (3) time and (4) location of slab deformation between video frames. After determining the location and timing of slab deformation, we calculated the deformation distances and time difference from the crack initiation point (impact of the snowboarder) and time and accounted for spatial and temporal uncertainties. This allowed us to drive an estimation of the average crack propagation speed in the determined direction.

We used Lucas and Kanade’s optical flow algorithms^37^ to stabilize camera movements during the video clip to generate motion vectors of pixels around static terrain features like rocks and snow roughness around the avalanche release area between video frames. We used the inverse optical flow motion to move the video frames and stabilize the scene so that every point on the slope remained at the same pixel location throughout the video clip.

EVM is a method to enhance small colour changes in videos that are otherwise invisible to the naked eye^38^. This method comprises the following steps: it applies spatial decomposition on the video frames and applies a temporal filter to the frame sequence. Subsequently, the filtered band for the targeted frequency range is amplified by a predetermined factor. Finally, the amplified downsampled bands are added to the original signal, and then the pyramid is collapsed to create the magnified signal. We compared the frequency bands in areas where the avalanche was released to areas where it was not released to filter out frequencies associated with noise. We used frequencies between 1.5 and 4.3 Hz and an amplifying colour magnification factor of 50.

We used the difference in pixel intensity between two consecutive frames to detect the slab deformation’s start time and location during the avalanche release. To filter out noise, we considered only areas where the pixel intensity changes between frames exceeded the changes in areas where the avalanche did not release. The coordinates of the video’s points of interest were obtained using a digital elevation model with a 0.25-m resolution and by matching key points of the slope (ridge and rocks, Extended Data Fig. 5a). We estimated the error of crack initiation time and the onset of slab deformations within one frame with a mean error of 0.02 s and a standard deviation of 0.01 s. We estimated the distance measurement error due to spatial decomposition in the EVM processing and the conversion from video frames to latitude/longitude coordinates to be, on average, 0.46 m, with a standard deviation of 0.07 m.

Our procedure to evaluate the crack propagation speed based on the EVM method was validated by applying it to (1) 2D and 3D numerical simulations performed on flat and inclined slopes (Extended Data Fig. 6) and (2) classical PST experiments on both flat and inclined slopes and a flat 5-m-long PST^31^ (Extended Data Fig. 7).

The density of the slab was estimated separately using two techniques: (1) based on the hand hardness and grain size obtained from a manual snow profile performed one day after the avalanche at Hohsaas station in Saas Grund (VS) at a distance of 10 km from the avalanche event; (2) based on a snow cover simulation (SNOWPACK^39^) and meteorological data available at the Orzival IMIS meteorological station, around 3 km from the avalanche. The manual snow profile is provided in Extended Data Fig. 5b. Based on ref. ^40^, an average slab density of 250 kg m^−3^ (±40 kg m^−3^) was evaluated. The SNOWPACK simulation gives an average slab density for the lowest-depth hoar weak layers of ~250 kg m^−3^, in good agreement with the estimation based on the manual snow profile. For the sake of computing the slab elastic modulus and elastic wave speeds, a slab density of 250 kg m^−3^ was thus chosen.

### Elastic wave speeds in snow

The mechanical properties of snow are time-dependent, and with increasing strain rates the strength decreases while the elastic modulus increases (for example, ref. ^41^). In addition, the ice matrix in snow has a highly disordered, cohesive-granular microstructure. As such, snow microstructure plays a crucial role in the rich rate- and temperature-dependent behaviour observed in snow (for example, refs. ^41,42^). It is known that density alone is not sufficient to describe snow mechanical properties, as, for a given density, values scatter by orders of magnitude due to differences in snow microstructure (for example, ref. ^43^). Furthermore, in slab avalanche release, the snow slab is usually composed of multiple layers with different snow types. To model the complete physics of snow slab avalanche release would thus require accounting for temperature, strain rate, snow density and microstructure. As many of the processes in snow mechanics are still poorly understood, the slab is therefore usually modelled as a homogeneous layer with a bulk density and an effective elastic modulus^25^. Therefore, to compute the speed of the elastic waves in snow, we used an approximation for the effective elastic modulus of the slab based on density according to the laboratory experiments of Scapozza^44^, who provides values similar to those measured in PST experiments^25^. The following power-law relationship was used:

2 $${E}={5.07}\times {10}^{9}{\left({\frac{\rho }{{\rho }_{{{{\rm{ice}}}}}}}\right)}^{5.13}$$

with *ρ*_ice_ = 917 kg m^−3^. In addition, we used a representative Poisson’s ratio^45^ of *ν* = 0.3.

### Numerical model and simulation set-up

The MPM is a hybrid Lagrangian–Eulerian numerical technique in which the Lagrangian particles track history-dependent variables such as position, velocity and deformation gradient, and the Eulerian grid enables computation of the spatial gradients of these quantities. The transfer of information between the grid and the particles is then handled by an interpolation scheme. The time is discretized using a symplectic Euler time integrator. Details of the applied algorithm are available in refs. ^10^ and ^46^. This algorithm is particularly suited to handling large topological changes and allows us to resolve the governing equations of continuum mechanics (equations (3) and (4)), where *ρ* is density, *t* is time, ***v*** is velocity, ***σ*** is the Cauchy stress tensor and ***g*** is gravitational acceleration:

3 $${\frac{D\rho }{Dt}}+{\rho \nabla }\cdot {{\boldsymbol{v}}}={0}$$

4 $${\rho }{\frac{D{{\boldsymbol{v}}}}{Dt}}={\nabla }\cdot {{\boldsymbol{\sigma }}}+{{\rho }}{{\boldsymbol{g}}}.$$

In the scope of finite strain elastoplasticity, the Cauchy stress ***σ*** can be related to the strain through an elastoplastic constitutive law, where ***F***^E^ denotes the elastic part of the deformation gradient ***F***:

5 $${{\boldsymbol{\sigma }}}=\frac{1}{\det {{\boldsymbol{F}}}}{\frac{\partial {{\varPsi }}}{\partial {{{\boldsymbol{F}}}}^{\rm{E}}}}{{{\boldsymbol{F}}}}^{{\rm{E}}^{\rm{T}}},$$

where $\varPsi$ is the elastoplastic potential energy density related to the Lamé coefficients based on the St. Venant–Kirchhoff hyperelastic model. A similar constitutive model to the one proposed in ref. ^10^ is used in this study to simulate the behaviour of both the slab and the weak layer. It is based on an associative cohesive Cam clay (CCC) yield surface and a softening–hardening law that depends on the material type (that is, weak layer, slab). The yield surface is expressed as

6 $${y(p,\,q)}={q}^{2}{({1}+{2\beta })}+{M}^{2}{({p}+{\beta }{p}_{0})({p}-{p}_{0})},$$

where *p* is the pressure, defined as *p* = tr(***τ***)/*d* (***τ*** is the Kirchhoff stress tensor and *d* is the dimension), *q* is the von Mises equivalent stress defined as ${q}={\sqrt{3/2{{\boldsymbol{s}}}:{{\boldsymbol{s}}}}}$, ***s*** = ***τ*** + *p****I*** is the deviatoric stress tensor, ***I*** is the identity matrix, *p*_0_ is the compressive strength, *βp*_0_ the tensile strength and *M* is the slope of the material critical state line without cohesion. For the slab, ${p}_{0}={p}_{0}^{{{{\rm{slab}}}}}$ may increase (hardening) or decrease (softening) depending on the amount of plastic deformation, according to equation (7):

7 $${p}_{0}^{{{{\rm{slab}}}}}={k}_{1}{\sinh }({\max }(-{{\epsilon }_{\rm{v}}^{\rm{P}}},\,{0})),$$

where *k*_1_ is the bulk modulus associated with a Young’s modulus of 1 MPa (kept constant in this study), and ${\epsilon }_{\rm{v}}^{\rm{P}}$ is the volumetric plastic deformation. For the weak layer, ${p}_{0}={p}_{0}^{{{{\rm{wl}}}}}$ is defined according to

8 $${p}_{0}^{{{{\rm{wl}}}}}={k}_{1}{\sinh }{({\max }({-\eta },\,{0}))},$$

where *η* is the anticrack plastic strain defined in ref. ^10^ according to

9 $${\dot{\eta }}=\left\{\begin{array}{l}{\alpha }\,| {\dot{\epsilon }}_{\rm{v}}^{\rm{P}}| ,\quad {{{\rm{if}}}}\,{t}\le {{t}_{\rm{c}}}\\ {\xi }\,{\dot{\epsilon }}_{\rm{v}}^{\rm{P}},\;\;\,\quad {{{\rm{if}}}}\,{t} > {{t}_{\rm{c}}}\end{array}\right.$$

for the weak layer. In the above equations, *t*_c_ is the time corresponding to complete softening, that is, *p*_0_ = 0, the time at which *β* is set to zero, making the behaviour of the weak layer cohesionless. *α* is a softening coefficient, and *ξ* is a hardening coefficient. The main difference with the weak-layer model developed by Gaume et al.^10^ lies in the fact that the softening and the hardening behaviour are now independent of each other. In addition, the constant prefactor *k*_1_ allows us to control the amount of volumetric collapse based on *ξ* only, independently of the elastic parameters. Hence, the collapse height of the weak layer *h* can be analytically predicted based on equations (10) and (9) as a unique function of *ξ* as follows:

10 $${\frac{h}{{D}_{{{{\rm{wl}}}}}}}={1}-{\exp }{\left({\epsilon }_{\rm{v}}^{\rm{P}}\right)}={1}-{\exp }{\left({\frac{1}{\xi }}{\sinh }^{-1}{\left({\frac{{p}_{0}}{{k}_{1}}}\right)}\right)}.$$

**Extended Data Fig. 9:**
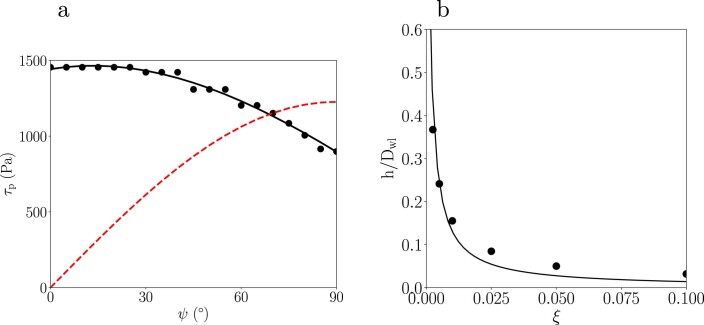
Mechanical behavior of the weak layer. **a**, Shear strength *τ*_p_ of the simulated weak layers as a function of slope angle *ψ* (black dots), fitted value (black line) and static shear stress *τ*_g_ (dashed red line). **b**, Relationship between relative collapse height and hardening coefficient.

Simulation results for different values of *ξ* are shown in Extended Data Fig. 9b, together with their analytical counterparts.

The numerical set-up consists of a PST with a bilayer system composed of a cohesive elastic slab and an elastoplastic weak layer (respectively 50 and 12.5 cm thick). The thickness of the weak layer was chosen in the higher range of measured values in PST experiments with full propagation (typically between 0.2 and 30 cm thick). We also performed additional MPM simulations with different weak-layer thicknesses (as low as 2 cm) to verify this did not affect the presented results. A crack of length *a* is created in the weak layer by a virtual saw (which sets the elastic modulus of particles in its neighbourhood to zero) until it propagates spontaneously once a critical crack length *a*_c_ is reached. The saw advances at a velocity of 1 m s^−1^, a speed that was verified to be sufficiently low not to affect the presented results. We used the MPM to perform 2D simulations of PSTs with length ranging from 25 to 140 m. This length was varied to ensure a steady crack propagation regime amongst all our simulations. The constitutive models previously described were used for the slab and the weak layer. The positions of the particles at the bottom of the weak layer are fixed. Extended Data Table 1 shows a synthesis of the parameter values used in this study. To evaluate the shear strength of the material for a given nominal load state (see below), we performed additional simulations for loading angles from 0 to 180°. This allowed us to obtain the weak-layer shear strength as a function of the normal stress. Based on the critical state line concept, the friction angle *ϕ* of the damaged weak layer can be directly related to the slope of the critical state line *M* following equation (11) and is equal to 21°, which is slightly lower in the exposed simulations than the one reported in ref. ^4^:

11 $${\phi }={\arcsin }{\frac{3M}{{6}+{M}}}.$$

Quantities of interest, such as the positions (*x*), the Kirchoff stress tensor (***τ***) and the volumetric plastic strain (${\epsilon }_{\rm{v}}^{\rm{P}}$) of each particle are outputted. We can then track the crack length (*a*), which is defined as the *x* position of the furthest plasticized particle. In parallel, the longitudinal evolution of the collapse height (*h*) and of the Kirchhoff stress tensor components (*τ*_*ij*_) of both the slab and weak layer are computed by averaging them on slices with a width of d*l* = 5 d*x*. The crack tip is defined as the location of the furthest plasticized particle (that is, the furthest particle presenting a plastic deformation). The computation of the crack propagation speed (${\dot{a}}$) is directly obtained from the longitudinal evolution of the crack tip position. The critical crack length (*a*_c_) is computed as the position of the saw for which the crack starts to self-propagate over a length larger than 10 cm ahead of the saw. The supercritical crack length (*a*_sc_) is evaluated from the crack propagation speed profile in a three-step approach. First, we search the location of the maximum local variation of the crack speed over a moving average of four frames according to a rolling median algorithm, which allow us to detect the acceleration of the propagation following the first peak. Based on this detection, the domain is resized and the algorithm is applied a second time to detect the ascending side of the peak, which allows a second resize of the domain. This enables us to evaluate a suitable temporal window for the search of the supercritical crack length *a*_sc_. Third, *a*_sc_ is estimated as the position corresponding to the maximum jerk (time derivative of the acceleration) within this window. The asymptotic crack propagation speed is computed from the average speed at the end of the propagation over a distance of 5*Λ*, ensuring that the propagation reaches a permanent regime.

### Analytical model for the onset of supershear fracture

After the supershear transition, the crack propagates in mode II (Fig. 1). We thus describe the onset of supershear transition based on a shear band propagation model to predict the supercritical crack length. The approach used is similar to that in refs. ^29,47,48^, but introduces the effect of the collapse height on the residual shear friction.

As a first approximation, we neglect inertia. We neglect the size of the process zone and the effect of slab bending. We assume continuity of displacements at the slab–weak layer interface, as well as homogeneous shear of the weak layer in the *y* direction. In addition, once failed, the weak layer has a constant residual shear strength ${\tau }_{\rm{r}}^{* }$ that depends on the collapse height. For a crack of length *a* with a tip located in *x* = 0 (Extended Data Fig. 2), it is possible to relate the stress field *τ*(*x*) to the displacement field *u*(*x*) as

12 $${\tau (x)}=\left\{\begin{array}{ll}{\tau }_{\rm{r}}^{* }\quad &\forall {x} < {0}\\ -{G}_{{{{\rm{wl}}}}}{\frac{u(x)}{{D}_{{{{\rm{wl}}}}}}}\quad &\forall {x}\ge {0} \end{array}\right.,$$

where *G*_wl_ and *D*_wl_ are the weak-layer shear modulus and thickness, respectively. Considering the force balance in the *x* direction of an elementary volume of length d*x*, it is possible to derive the local force balance acting on this local slab section of density *ρ* and thickness *D* as

13 $${\frac{{\rm{d}}N}{{\rm{d}}x}}+{\tau (x)}-{\tau }_{\rm{g}}={0},$$

in which *τ*_g_ = *ρgD*sin*ψ*, and *N* is the slab tensile force given by the plain strain elastic relationship ${N(x)}={E}^{\prime} {D}{\frac{{\rm{d}}u}{{\rm{d}}x}}$, with *E*′ = *E*/(1 − *v*^2^), leading to the following second-order equation:

14 $${\frac{{\rm{d}}^{2}u}{{\rm{d}}{x}^{2}}}-{\frac{u(x)}{{{{\varLambda }}}^{2}}}-{\frac{{\tau }_{\rm{g}}}{E^{\prime} D}}={0}\,{\forall {x}\ge {0}}$$

with ${{\varLambda }}={\sqrt{\frac{{E}^{\prime} {D}{D}_{{{{\rm{wl}}}}}}{{G}_{{{{\rm{wl}}}}}}}}$, and which has the well-known general solution

15 $${u(x)}={c}_{1}{\rm{e}}^{x/{{\varLambda }}}+{c}_{2}{\rm{e}}^{-x/{{\varLambda }}}-{\frac{{\tau }_{\rm{g}}}{{E}^{\prime}{D}}}{{{\varLambda }}}^{2}.$$

The two constants *c*_1_ and *c*_2_ can be evaluated from boundary conditions. First, tension in the slab far from the crack vanishes, which leads to *c*_1_ = 0. Second, we assume a constant and homogeneous effective residual shear stress, which allows us to compute slab tension at the crack tip, that is, ${N}{(x=0)}={({\tau }_{\rm{g}}-{\tau }_{\rm{r}}^{* }){a}}$, leading to ${c}_{2}=-{{\varLambda }}{\frac{({\tau }_{\rm{g}}-{\tau }_{\rm{r}}^{* }){a}}{{E}^{\prime}{D}}}$. As a consequence, one can obtain the profile of the weak-layer shear stress as

16 $${\tau (x)}=\left\{\begin{array}{ll}{\tau }_{\rm{g}}{\left({1}+{\frac{a}{{{\varLambda }}}}{\left({1}-{\frac{{\tau }_{\rm{r}}^{* }}{{\tau }_{\rm{g}}}}\right)}{\rm{e}}^{-\frac{x}{{{\varLambda }}}}\right)}\quad &\forall {x}\ge {0}\\ {\tau }_{\rm{r}}^{* }\quad &\forall {x} < {0}\end{array}\right..$$

One can thus determine the supercritical crack length *a*_sc_ based on the shear stress at the crack tip (*x* = 0) and the shear strength of the weak layer in mode II *τ*_p_ as

17 $${a}_{\rm{sc}}={{\varLambda }}{\frac{{\tau }_{\rm{p}}-{\tau }_{\rm{g}}}{{\tau }_{\rm{g}}-{\tau }_{\rm{r}}^{* }}},\quad {\tau }_{\rm{g}}={\rho gD\sin \psi },\quad {\tau }_{\rm{r}}^{* }={\rho gD\cos \psi \tan {\phi }^{* }}.$$

The shear strength (which depends on the nominal load) in mode II of the simulated weak layer is shown in Extended Data Fig. 9a as a function of slope angle. The loss of contact during weak-layer collapse results in an effective weak-layer friction angle $\phi^*$ ≤ *ϕ*. The value of $\phi^*$ is obtained by fitting equation (17) to simulation results for different collapse heights *h*, which leads to the relation between ${\phi }^{* }$ and *h* shown in Fig. 3. In turn, as the effective residual stress is a function of the effective friction angle $\phi^{*}$, ${\tau }_{\rm{r}}^{* }$ also depends on the collapse height *h*.

## Online content

Any methods, additional references, Nature Research reporting summaries, source data, extended data, supplementary information, acknowledgements, peer review information; details of author contributions and competing interests; and statements of data and code availability are available at 10.1038/s41567-022-01662-4.

### Supplementary information

Supplementary Video 1 **Effect of slope angle on crack propagation dynamics**. Crack tip morphology and dynamics for two MPM simulations (top, *ψ* = 0°; bottom, *ψ* = 30°). The colourmap of the slab represents the relative collapse height. Weak layer fracture is indicated in red.

Supplementary Video 2 **Video of the accidentally triggered snow slab avalanche**. Complete video of the release and flow of the snow slab avalanche analysed in this study.

Supplementary Video 3 **Analysis of the avalanche video**. Frame by frame analysis of the change in pixel intensity from the avalanche video.

Supplementary Video 4 **Effect of slab fractures on crack propagation dynamics**. Crack tip morphology and dynamics for two MPM simulations (top, *σ*_t_ = 6 kPa; bottom, *σ*_t_ = 11 kPa). The colourmap of the slab represents the relative collapse height. Weak layer and slab fracture are indicated in red.

Supplementary Video 5 **Centered propagation saw test simulations**. Crack tip morphology and dynamics for two MPM simulations of a centered propagation saw test (top, *ψ* = 0°; bottom, *ψ* = 30°). The colourmap of the slab represents the relative collapse height. Weak layer fracture is indicated in red.

## Figures and Tables

**Extended Data Table 1:**
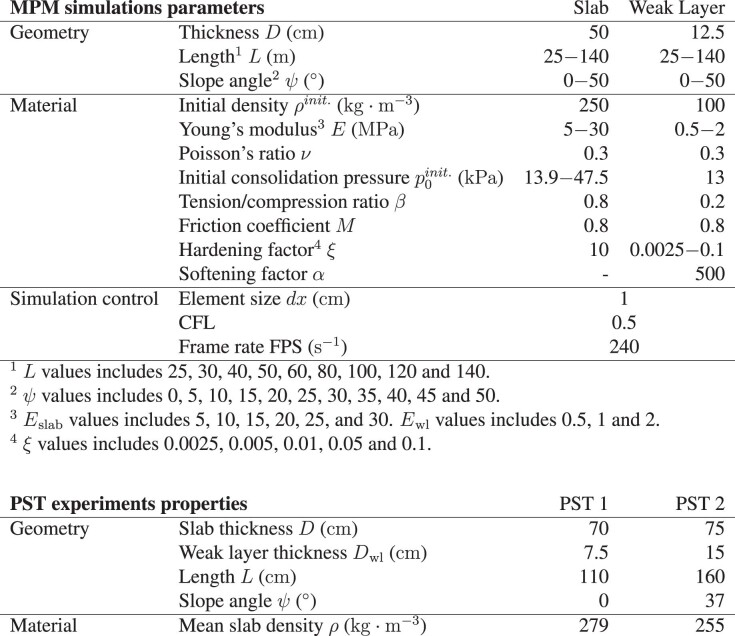
Numerical and experimental parameters

Top: Parameters values employed in MPM simulations. Bottom: Geometrical and mechanical properties of the Propagation Saw Test experiments.

## Data Availability

All data needed to evaluate the conclusions in the paper (PST, avalanche and simulation data) are present in the manuscript and are also available at 10.5281/zenodo.6488738.

## Code availability

The MPM numerical model used in this study can be found in a previous publication at https://www.nature.com/articles/s41467-018-05181-w.
